# The Therapeutic Potential of Anthocyanins: Current Approaches Based on Their Molecular Mechanism of Action

**DOI:** 10.3389/fphar.2020.01300

**Published:** 2020-08-26

**Authors:** Bahare Salehi, Javad Sharifi-Rad, Francesca Cappellini, Željko Reiner, Debora Zorzan, Muhammad Imran, Bilge Sener, Mehtap Kilic, Mohamed El-Shazly, Nouran M. Fahmy, Eman Al-Sayed, Miquel Martorell, Chiara Tonelli, Katia Petroni, Anca Oana Docea, Daniela Calina, Alfred Maroyi

**Affiliations:** ^1^Noncommunicable Diseases Research Center, Bam University of Medical Sciences, Bam, Iran; ^2^Student Research Committee, School of Medicine, Bam University of Medical Sciences, Bam, Iran; ^3^Phytochemistry Research Center, Shahid Beheshti University of Medical Sciences, Tehran, Iran; ^4^Dipartimento di Bioscienze, Università degli Studi di Milano, Milano, Italy; ^5^Department of Internal Medicine, University Hospital Centre Zagreb, School of Medicine, University of Zagreb, Zagreb, Croatia; ^6^Faculty of Allied Health Sciences, University Institute of Diet and Nutritional Sciences, The University of Lahore, Lahore, Pakistan; ^7^Department of Pharmacognosy, Faculty of Pharmacy, Gazi University, Ankara, Turkey; ^8^Department of Pharmacognosy, Faculty of Pharmacy, Ain-Shams University, Cairo, Egypt; ^9^Department of Pharmaceutical Biology, Faculty of Pharmacy and Biotechnology, German University in Cairo, Cairo, Egypt; ^10^Department of Nutrition and Dietetics, Faculty of Pharmacy, University of Concepcion, Concepcion, Chile; ^11^Unidad de Desarrollo Tecnológico, Universidad de Concepción UDT, Concepcion, Chile; ^12^Department of Toxicology, University of Medicine and Pharmacy of Craiova, Craiova, Romania; ^13^Department of Clinical Pharmacy, University of Medicine and Pharmacy of Craiova, Craiova, Romania; ^14^Department of Botany, University of Fort Hare, Alice, South Africa

**Keywords:** anthocyanins, biodisponibility, neuroprotective, cardioprotective, antiobesity, antidiabetic, antioxidant, anticancer

## Abstract

Anthocyanins are natural phenolic pigments with biological activity. They are well-known to have potent antioxidant and antiinflammatory activity, which explains the various biological effects reported for these substances suggesting their antidiabetic and anticancer activities, and their role in cardiovascular and neuroprotective prevention. This review aims to comprehensively analyze different studies performed on this class of compounds, their bioavailability and their therapeutic potential. An in-depth look in preclinical, *in vitro* and *in vivo*, and clinical studies indicates the preventive effects of anthocyanins on cardioprotection, neuroprotection, antiobesity as well as their antidiabetes and anticancer effects.

## Introduction

Anthocyanins (ACNs) are natural bioactive water-soluble phenolic compounds, which represent one of the principal families of natural pigments (orange, red, violet, and blue colors) (Riaz et al., 2016). More than 700 ACNs were identified in nature, and they are produced by plants to attract insects to flowers for pollination and herbivorous animals to fruits for seed dissemination, as well as for the protection of plant cells against UV radiation damage (Markakis, 2012; Wallace and Giusti, 2013; Warner, 2015). ACNs are widely found in different plant families, for example, *Vitaceae*, *Rosaceae*, *Ericaceae*, *Saxifragaceae*, *Caprifoliaceae*, *Cruciferae*, and *Fabaceae* (Mazza and Miniati, 2018). The name is derived from the Greek word *anthos*, which means ﬂower and *kyanos* (blue) (Sui, 2016). ACNs are a subclass of flavonoids and are distributed in different parts of the plants, especially in flowers and fruits (Gupta, 2006; Pascual-Teresa et al., 2010; Wallace and Giusti, 2013; Riaz et al., 2016).

ACNs are natural bioactive compounds with many pharmacological effects: antioxidant, antiinflammatory, prevention of age-related chronic diseases: cardiovascular disease (CVD), cancers, neurodegenerative, and eye-related diseases. ACNs also have antiviral properties. Recent *in vitro* studies have shown that they can inhibit the replication of viruses such as herpes simplex, parainfluenza virus, syncytial virus, HIV, rotavirus, and adenovirus (Pour et al., 2019).

The broad spectrum of pharmacological properties supported by preclinical and clinical evidence, associated with a low toxicity make their pharmacotherapeutic use very attractive.

ACNs are used in the food industry to replace synthetic colorants (Mazza and Miniati, 2018).

The current review comprehensively analyzes different studies performed on this class of compounds, their bioavailability, the therapeutic potential, molecular mechanisms of action, as well as their clinical significance in the prevention of chronic noncommunicable diseases.

## Modern Technology of Extraction, Stability, and Bioavailability of ACNs

### Extraction of ACNs

ACNs are a group of phytochemicals that are evaluated as important compounds in nutrition and medicine. As plant metabolites, they are known to be beneficial to health by acting as antioxidants and having antiinflammatory effects. Due to these beneficial effects on health, flavonoids are used in nutraceutical, pharmaceutical, medicinal and cosmetic production. ACNs molecules are found in a variety of fruits, vegetables, grains, roots, stems, leaves, flowers, and bark. In order to process ACNs as nutritional supplements, pharmaceuticals or as active ingredients in food or cosmetics, ACNs must be released from the plant cell matrix.

Therefore, a strong, efficient, and reliable extraction method is needed.

Ultrasonic extraction is a nonthermal insulation technique that prevents thermal decomposition of heat sensitive compounds. Ultrasound extraction promotes the release of high quality ACNs from plants resulting in higher yields and a faster process. Sonicare is a light, green and efficient technique for the industrial production of food ACNs (Belwal et al., 2019)

Advantages of ultrasound extraction: higher yields, fast extraction process: in a few minutes high quality extracts—easy, nonthermal extraction green solvents (water, ethanol, glycerin, vegetable oils, etc.), easy and safe operation, low operating, and investment costs, robustness and low maintenance.

Enzymatic methods are more efficient because they offer the possibility of being exploited high regioselectivity of these biocatalysts, achieving a selective functionalization of the flavonoid.

### Stability of ACNs

Therapeutic use of ACNs is limited by reduced stability and their low solubility, both in organic solvents and in aqueous solutions. However, ACNs can be converted to glycosylated or acylated derivatives by chemical, enzymatic or chemoenzimatics methods.

The transformation of ACNs into bioconjugates by fatty acid acylation offers the possibility to introduce into the molecule another biologically active function and thus positively modify not only physical properties such as solubility, but and biological activity.

The protection and especially the controlled release of various organic molecules is achieved almost exclusively by means of encapsulated compounds. For example, cyclodextrins are part of the most used class receptors in host-guest inclusion chemistry. Advantages of encapsulation in cyclodextrins of active substances biological effects are: improving bioavailability, increasing stability, reducing side effects.

### Bioavailability of ACNs

The chemical structure of ACNs is composed of an aglycone basic unit (polyhydroxy and polymethoxy derivatives of 2-phenylbenzopyrylium or flavylium salts) and a glycone, the sugar moiety. The position, nature and number of sugar moieties and their acylation as well as the position and number of hydroxyl groups and their degree of methylation result in various types of ACNs (Jackman et al., 1987; Mazza and Miniati, 2018).

**Figure 1:**
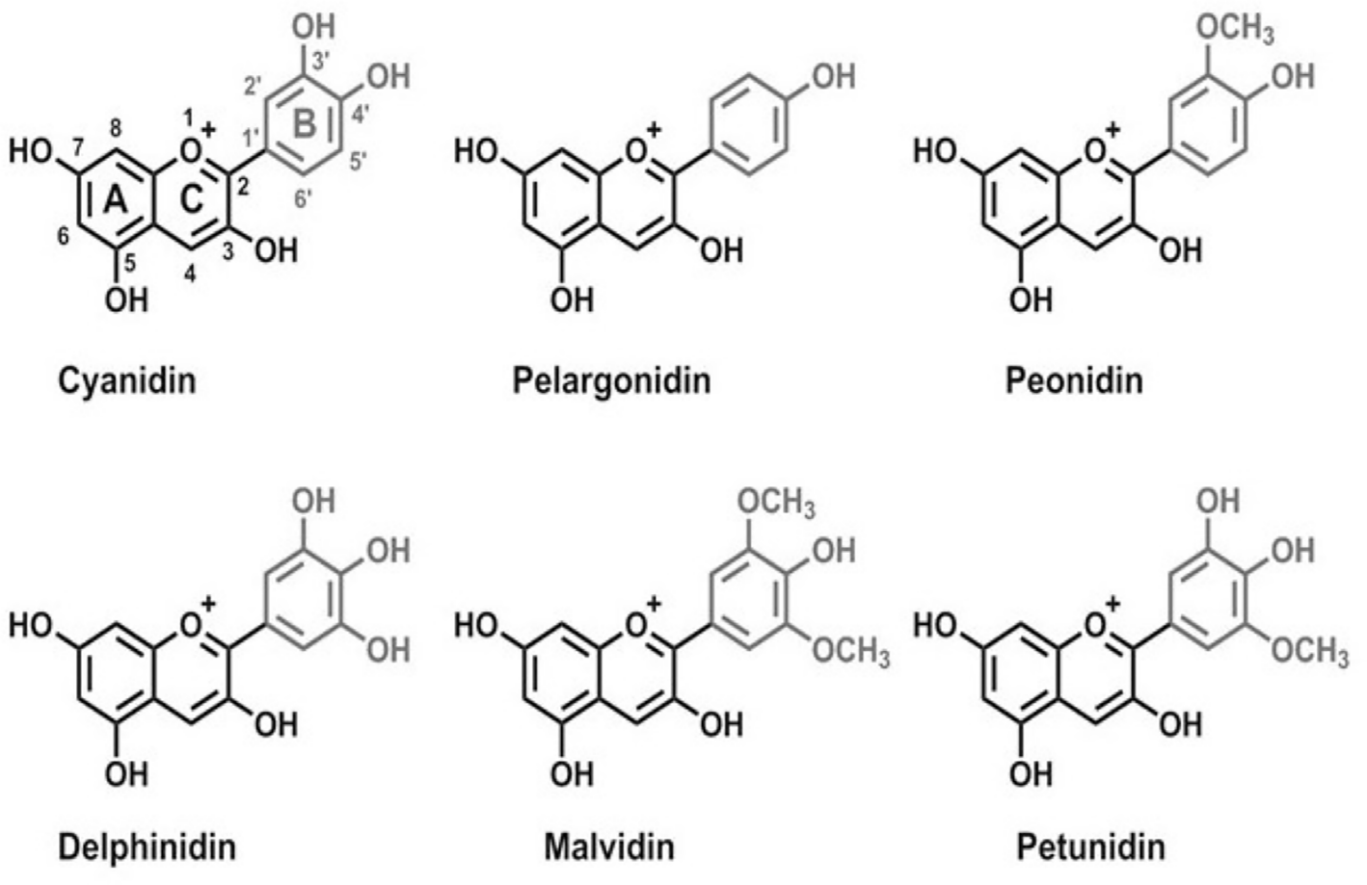
Chemical structures of main anthocyanins: pelargonidin, cyanidin, peonidin, delphinidin, malvidin, and petunidin.

Pelargonidin, delphinidin, cyanidin, peonidin, petunidin, and malvidin (**Figure 1**) are the most frequently occurring anthocyanidins in plants. Similar to other flavonoids, ACNs are characterized by the same carbon skeleton (C6-C3-C6). Acylated ACNs consist of an additional organic acid unit usually bonded to the sugar at the C-3 position (Markakis, 2012).

The oral bioavailability of ACNs is poor and it is correlated with their stability, lack of site-specificity in distribution, rapid elimination and clearance from the body, and the dietary source of the compound and food matrix interactions (Milbury et al., 2010).

Gastric administration of ACNs in rats demonstrated that they are initially absorbed in the stomach (about 25%) through a bilitranslocase-mediated mechanism and can be detected in their native form in plasma within 6 min. In contrast, they can be found as methylated and conjugated derivatives in bile within 20 min, suggesting that ACN metabolites are rapidly formed in the liver and eliminated *via* bile, but not distributed in blood (Passamonti et al., 2003; Talavera et al., 2003).

ACNs are also rapidly absorbed (12%–15%) in the small intestine of rats and found in their natural form and methylated derivatives in plasma within 25 min, but also excreted into bile and urine as intact glycosides or methylated/glucuronidated derivatives (Talavera et al., 2004). Absorption of ACNs in the small intestine may occur by deglycosylation to aglycones followed by passive transport through intestinal epithelium (Kay, 2006) or by an active transport mechanism using intestinal sodium-dependent glucose transporter 1 (SGLT1) or bilintranslocase (Walton et al., 2006a; Passamonti et al., 2009).

Most ACNs in the blood are found in the form of their metabolites which have a lower pharmacological activity than the primary compounds. (Walton et al., 2006a; Passamonti et al., 2009).

The instability, high reactivity and low extraction possibility limit their potential applications of ACNs in food and pharmaceutical industries (Castañeda-Ovando et al., 2009).

## Pharmacological Activities of ACNs: An Overview on Molecular Mechanisms

### Neuroprotective Effects

**Figure 2:**
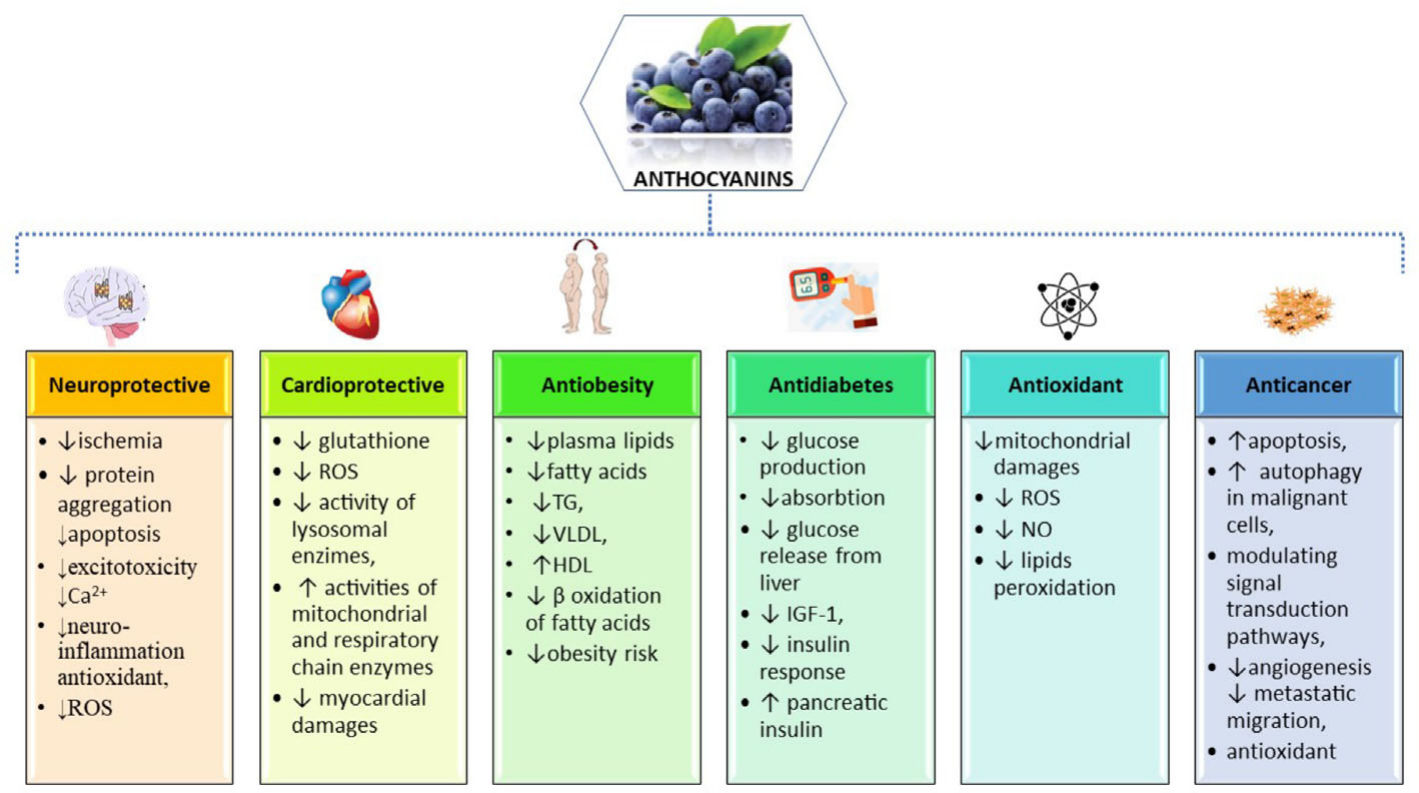
Summarized scheme of the most important pharmacological properties and molecular mechanisms of action of anthocyanins.

Due to its high demand of energy and high lipid content, the central nervous system (CNS), especially the brain, is particularly susceptible to excessive reactive oxygen species (ROS) (Nussbaum et al., 2017) (**Figure 2**). A high production of oxygen leads to a high production of intracellular ROS during cellular respiration within mitochondria (Salehi et al., 2019c). In addition, exogenous sources of ROS may be environmental pollution, smoking, unhealthy diet, UV-B radiation, drug metabolites and infections (Singh et al., 2019). In the CNS, a specific innate immune system, consisting of resting or activated glial cells, protects the nervous system against pathogens or injuries, but an excessive or prolonged inflammatory response may contribute to neuronal apoptosis and may facilitate the progression of neurodegenerative diseases (Russo and Mcgavern, 2015)

Many dietary ACNs contain multiple ACNs compounds with neuroprotective effects. Dietary ACNs from blue corn protected brain from the mitochondrial DNA common deletion (mtDNA CD) induced by a moderate ethanol consumption (Demeilliers et al., 2017). Finally, we showed that the administration of ACN-rich purple corn extract has a protective effect on the development of orofacial allodynia in an *in vivo* model of inflammatory trigeminal pain, and that it reduces trigeminal macrophage infiltration and microglial activation both *in vivo* and *in vitro* (Magni et al., 2018). The neuroprotective effect of purple corn is comparable to the antiinflammatory effects of acetyl salicylic acid, which does not modify microglia activation.

Therefore, a possible application of ACN-rich dietary supplements as co-adjuvant therapy to pharmacological treatment or as a preventive strategy against trigeminal pain, aimed at reducing drugs dosage and adverse effects might be proposed.

#### Perspectives on Pathophysiology of Neurodegenerative Disorders and Cerebral Ischemia

##### Neurodegenerative Disorders: A Brief Overview on Pathophysiology

These are a group of chronic diseases characterized by a progressive loss of neurons in brain or spinal cord, leading to a progressive impairment in cognitive and motor functions and ultimately resulting in severe disability (Calina et al., 2020; Sharifi-Rad et al., 2020b). Chronic neurodegenerative diseases include Alzheimer’s disease (AD), Parkinson’s disease (PD), and amyotrophic lateral sclerosis (ALS) (Jellinger, 2009).

Neurodegenerative disorders and cerebral ischemia are associated to three common factors triggering the onset of neuronal apoptosis, (Jellinger, 2009; Li et al., 2019) which is the main mechanism responsible for neuron loss:

- oxidative stress
- excitotoxicity
- neuroinflammation

Oxidative stress is due to a significant reduction in the ability of neuronal cells to scavenge excessive ROS, leading to oxidative damage of macromolecules (i.e. DNA oxidation, protein carbonylation, and lipid peroxidation) and to mitochondrial dysfunction, additional generation of ROS and neuronal apoptosis (Mariani et al., 2005; Singh et al., 2019). Increased oxidative stress and mitochondrial dysfunction precede the deposition of neurotoxic amyloid-β (Aβ) protein aggregates, a typical hallmark of AD (Salehi et al., 2020a). They also cause the formation of the Lewy bodies, insoluble inclusions mainly consisting of damaged α-synuclein, associated with neuron loss and dopamine deficiency in the substantia nigra of PD patients (Wang et al., 2014; Puspita et al., 2017).

Excitotoxicity is a common pathogenic factor in neurodegenerative diseases (Dong et al., 2009; Mattson, 2019). Excitotoxicity consists of the overstimulation of glutamate receptors [i.e., N-methyl-D-aspartic acid (NMDA) and α-amino-3-hydroxy-5-methyl-4-isoxazolepropionic acid (AMPA)], causing an excessive influx of calcium ions from the extracellular space (Dong et al., 2009; Mattson, 2019). This calcium overload triggers intracellular signaling cascades, leading to mitochondrial depolarization, increased ROS and nitric oxide (NO) production, degradation of macromolecules and ultimately apoptosis, thus indicating the existence of a link between excitotoxicity and oxidative stress (Dong et al., 2009; Mattson, 2019). Upregulation of NMDA receptors has been associated to Aβ deposition in AD (Parameshwaran et al., 2008), whereas an overstimulation of AMPA receptors with kainic acid in rats specifically promoted loss of motor neurons, which are particularly rich in AMPA receptors, thus suggesting a specific role of excitotoxicity in ALS (Sun et al., 2006).

Neuroinflammation may be also triggered as a response to the aberrant deposition of protein aggregates, such as Aβ in AD, α-synuclein in PD and DNA-binding protein-43 in ALS (TDP-43). (Zhang et al., 2005; Stewart et al., 2010; Van Langenhove et al., 2012).

##### Cerebral Ischemia: A Brief Overview on Pathophysiology

This is a condition in which the brain does not receive enough blood to meet its metabolic needs. Thus, the resulting lack of oxygen can cause the death of brain tissue and therefore an ischemic stroke (Tsatsakis et al., 2019). Cerebrovascular ischemia causes loss of neurons in localized regions of brain by mechanisms similar to those in neurodegenerative diseases (Chen et al., 2011).

Oxidative stress caused by reperfusion following cerebrovascular ischemia triggers the production of excessive superoxide radicals by mitochondria with increasing ROS and mitochondrial dysfunction. These superoxide radicals combined with NO, produced by ischemia-induced neuronal NO synthase (nNOS), generate reactive nitrogen species (i.e. peroxynitrite) that further damage neuronal cellular proteins by nitrosylation (Eliasson et al., 1999).

Excitotoxicity is also a pathogenic factor in cerebrovascular ischemia. Massive release of presynaptic glutamate caused by cerebrovascular ischemia causes a consequent increase in NMDA post-synaptic receptors (Szydlowska and Tymianski, 2010; Li Y. et al., 2016) and finally loss-of-function mutations in the parkin gene (hereditary PD PARK2 gene) inducing a proliferation of glutamate post-synaptic receptors, thus causing a sensitization to excitotoxicity in the substantia nigra in PD (Helton et al., 2008).

Neuroinflammation in cerebral ischemia: neurons injured by cerebral ischemia release cytokines and chemokines, that activate resting microglial cells to secrete antiinflammatory neuroprotective cytokines, in order to assist in repairing neuronal cells, but also to promote the synthesis of proinflammatory cytokines (e.g,. IL-1β and TNF-α), iNOS and the production of NO, a reactive molecule which destroys invading pathogens (Di Filippo et al., 2010; Buga et al., 2019; Subhramanyam et al., 2019). In case of prolonged production, the inflammatory response may result in neuronal damage and finally in apoptosis (Block and Hong, 2007).

#### Mechanistic and Molecular Aspects of Neuroprotective Effects of ACNs

##### Neurodegenerative Diseases

These studies have reported the neuroprotective effect of ACNs in preclinical models of neurodegenerative diseases by multiple mechanisms (Tsuda, 2012; Ullah et al., 2019).

Antioxidant Mechanisms:

- The neuroprotective effect is accomplished because of rapid absorption of ACNs and their capacity to cross the blood brain barrier (BBB). Since they can reach the brain in their native form, ACNs can exert their antioxidant activity as direct scavengers of ROS (Shih et al., 2011; Casedas et al., 2018).
- They can also activate the antioxidant response by promoting the nuclear translocation of nuclear factor erythroid 2–related factor 2 (Nrf2) or by stimulating the activity of antioxidant enzymes, such as SOD, CAT and GPx (Casedas et al., 2018; Pacheco et al., 2018).

*In vitro* studies have demonstrated that ACNs prevent the intracellular calcium overload, thereby causing excitotoxicity and the progression of neurodegenerative diseases (Ye et al., 2010; Shih et al., 2011; Ullah et al., 2014; Badshah et al., 2015).

AntiNeuroinflammation Mechanisms:

- Inhibit the nuclear translocation of NF-κB, thereby preventing the activation of proinflammatory molecules, such as COX-2, iNOS, IL-1β, TNF-α (Kim et al., 2017).
- Reduce the intracellular signaling pathways of mitogen-activated protein kinases (MAPKs): c-Jun N-terminal kinase *(*JNK*)* and P38 mitogen-activated protein kinases (p-38 MAPK), also reducing the activation of proinflammatory cytokines (Amin et al., 2017).
- Antiinflammatory effect, by inhibiting the activity of COX-2 enzyme (Mulabagal et al., 2009).

Antiapoptotic Mechanisms:

- Prevent the release of apoptosis-inducing factor (AIF) from mitochondria and its migration into the nucleus, where it triggers DNA fragmentation by a caspase-independent pathway (Min et al., 2011),
- to increase the expression of the proapoptotic factor B cell lymphoma-2 (Bcl-2),
- to reduce the expression of the antiapoptotic factor B-cell lymphoma protein associated X (Bax) (Ali Shah et al., 2013; Khan M.s. et al., 2019).

These have proved that ACNs can prevent the onset and progression of neurodegenerative diseases as well as that they can improve learning and memory in aging model mice.

The first evidence of the neuroprotective effect of ACNs was obtained when 19 months-old aged rats (comparable to 60 years old humans) fed with blueberry extracts for 8 weeks showed a significant improvement in motor function in the accelerated rotarod test and effectively reversed age-related deficits in neuronal and cognitive function in a test of learning and memory, such as the Morris water performance (Joseph et al., 1999). More recently, similar results were obtained in galactose-induced aging models or aged rats fed with ACNs, showing a delayed age-related decline in spatial learning and memory (Andres-Lacueva et al., 2005; Rehman et al., 2017; Wei J. et al., 2017).

Concerning Alzheimer diseases (AD), the APP/PS1 transgenic mouse, carrying mutations in amyloid precursor protein (APP) and presenilin-1 (PS1), fed with blueberry extract from 4 months of age showed no deficits in Y-maze performance (at 12 months of age) with no difference in Aβ plaques compared to nontransgenic mice (Joseph et al., 2003). Since then, it was shown that dietary ACNs from mulberry extracts applied to a senescence-accelerated mouse model of AD (SAMP8) reduced Aβ plaques and improved learning and memory ability in avoidance response tests, by activating the Nrf2-dependent antioxidant defense system (Shih et al., 2010).

Using an Aβ-induced model of AD in rats, intragastrically applied ACNs from black soybean were shown to reverse Aβ-induced neuronal apoptosis by suppressing protein expression of intrinsic apoptotic pathway (Bax, cytochrome C, caspase-9 and caspase-3) (Badshah et al., 2015). More recently, dietary supplementation of ACNs from Korean black beans to the APP/PS1 mouse model of AD demonstrated that ACNs reduce oxidative stress induced by Aβ aggregation by activation of the p-PI3K/Akt/GSK3β pathway, which has been found to promote nuclear translocation of Nrf2 and the activation of HO-1 and glutathione cysteine ligase modulatory subunit (GCLM) target genes.

ACNs prevent apoptosis and neurodegeneration by suppressing the activation of caspase-3. As a result, memory-related pre- and post-synaptic protein markers and memory functions in both Morris water maze and the Y-maze tests were improved (Ali et al., 2018).

Concerning Parkinson Disease, an interesting epidemiological study highlighted that a regular intake of ACNs, based on consuming strawberries and blueberries, results in a significantly lower PD risk (Gao et al., 2012). In a MPTP-induced mouse model of PD, oral supplementation of ACNs from a mulberry extract showed a significant reduction in bradykinesia, in loss of dopaminergic neurons in substantia nigra and in depletion of dopamine depletion, related to a reduced expression of the proapoptotic Bax protein (Kim et al., 2010). Studies in rotenone-induced cell models of PD suggest that ACNs attenuate mitochondrial dysfunction by reducing the rotenone-induced damage of mitochondrial complex I of the electron transport system, and reduce neuroinflammation resulting from microglial activation, thus preserving dopaminergic neurons (Strathearn et al., 2014).

Only one study assessed the potential of ACNs in preventing the onset and progression of ALS, characterized by loss of motor neurons in brain, brainstem and spinal cord (Winter et al., 2018). Oral administration of an ACN-rich extract from strawberries in a mouse model of ALS, carrying a G93A mutation in the human SOD1 gene (hSOD1^G93A^), modestly but significantly delayed the onset of ALS (about 17 days) and extended the survival (about 11 days) when to untreated hSOD1^G93A^ mice, in which ALS onset occurred at about 90 days of age and progress to end-stage of disease at about 120 days age. Supplementation with ACNs significantly preserved grip strength and neuromuscular junctions in gastrocnemius muscle, but did not prevent motor neuron loss in spinal cord. On the other hand, a significant reduction in neuroinflammation (i.e. activated astrocytes) was observed in spinal cord (Winter et al., 2018).

Multiple sclerosis (MS) is a result of a neuronal demyelination process and not neuron loss, but shares some common pathological mechanisms with neurodegenerative diseases(Padureanu et al., 2019). Recent studies have highlighted that ACNs have a protective effect on the onset and progression of MS by reducing oxidative stress, neuroinflammation and the activity of ion pumps. (Carvalho et al., 2015).

ACNs reduced demyelination in a rat model of MS by restoring glutathione level and SOD activity, suggesting that a possible Nrf2-mediated antioxidant mechanism of protection may occur (Carvalho et al., 2015). In the same study, ACNs were found to reduce infiltration of inflammatory cells, the expression of proinflammatory cytokines, such as IL-1β and TNF-α, to increase the expression of antiinflammatory cytokines, like IL-10, and finally to increase the expression of Na^+^, K^+^-ATPase and Ca^2+^-ATPase, thus restoring neuronal functions (Carvalho et al., 2015).

Other neuroprotective effects of ACNs were recently demonstrated in animal models of neurotoxicity or inflammatory pain.

##### Cerebral Ischemia

**Figure 3:**
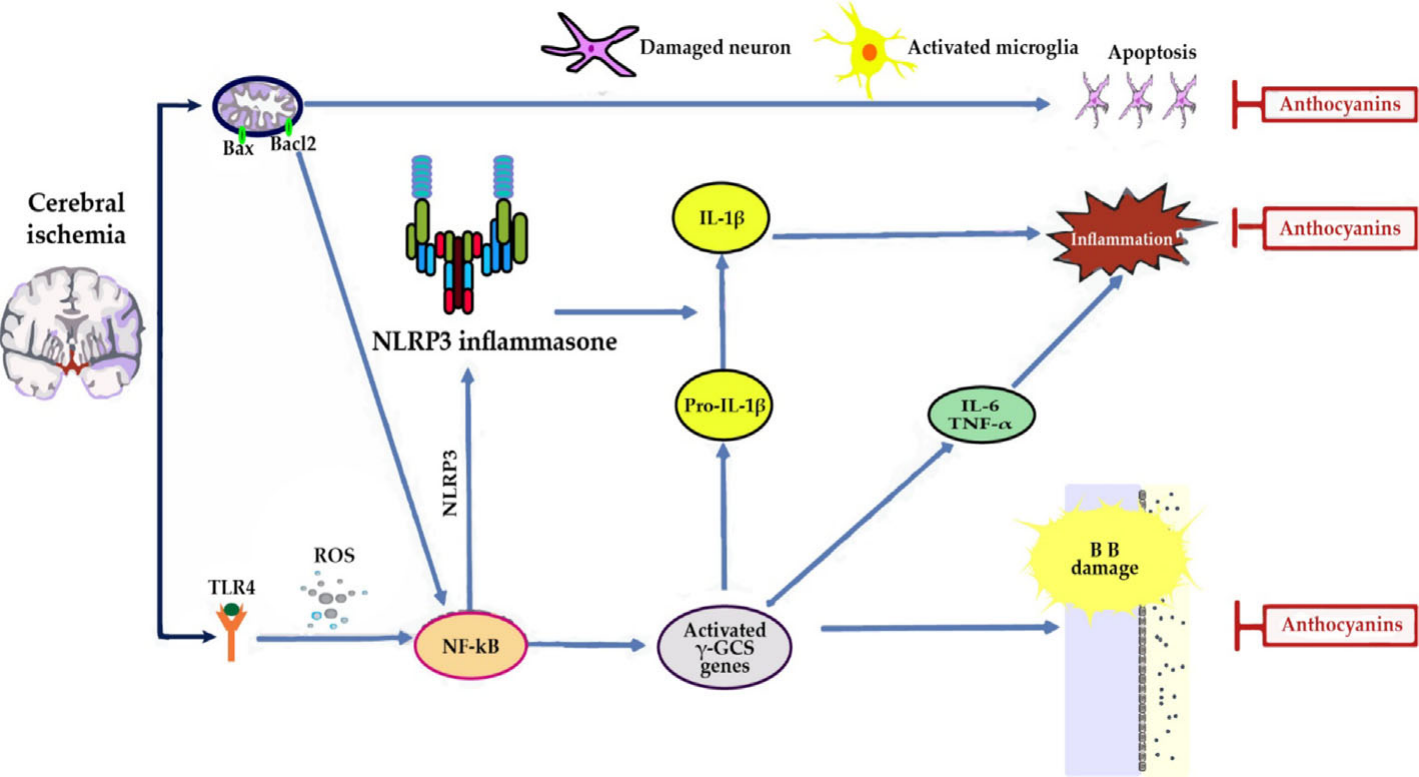
In cerebral ischemia, anthocyanins (ACNs) reduce neuroinflammation by: (i) decreasing the expression of Toll-like receptor 4 (TLR4), an activator of nuclear factor kappa B (NF-κB), and tumor necrosis factor-α (TNF-α) proinflammatory cytokine expression (Cui et al., 2018). (ii) Reducing the expression of inducible nitric oxide synthase (iNOS) and neuronal nitric oxide synthase (nNOS), targets of NF-κB, and as a consequence NO content at cerebral level, thus reflecting a reduction in brain damage (Di Giacomo et al., 2012; Cui et al., 2018). A significant increase of eNOS was observed which may result in vasorelaxation with a consequent attenuation of the ischemic insult and promotion of functional recovery of the ischemic zone (Di Giacomo et al., 2012). (ii) Direct antiapoptotic role, since they partially blocked AIF, but not cytochrome c release from mitochondria, thus indicating that guanine nucleotide exchange factor (C3G) reduced apoptosis by suppressing a caspase-independent pathway (Min et al., 2011).

Antioxidant defense in cerebral ischemia: ACNs promote activation of Nuclear factor erythroid 2-related factor 2 (Nrf2) and the consequent increase in the expression of the Heme oxygenase*-*1* (*HO*-*1*)* and γ-glutamyl cysteine synthase (γ-GCS) genes, contributing to decrease brain levels of superoxide and lipid peroxidation (Min et al., 2011; Di Giacomo et al., 2012; Cui et al., 2018) (**Figure 3**).

In addition, Nrf2 activation has been recently found to inhibit nod-like receptor protein 3 (NLRP3) inflammasome by regulating the thioredoxin1/thioredoxin interacting protein complex (TRX1)/TXNIP (Hou et al., 2018). This complex inhibits caspase-1, IL-6 and IL-1β activation, thereby inhibiting apoptosis and inflammatory responses and reducing brain damage (Cui et al., 2018; Padureanu et al., 2019; Mititelu et al., 2020).

These studies have shown the neuroprotective effect of ACNs in rat or mouse models of cerebral ischemia and defined some of the possible mechanisms of protection (Zhang et al., 2019).

Pre-treatment of middle cerebral artery occlusion (MCAo) rat models with orally administered high dose of ACNs (up to 300 mg/kg) significantly reduced cerebral infarct size (Shin et al., 2006; Cui et al., 2018). On the other hand, both pre-treatment (1h) and post-treatment (*i.e.* during reperfusion) with lower doses of purified C3G (2 mg/kg i.p administered or 10 mg/kg orally administered) also efficiently reduced cerebral infarct size in MCAo rat model (Min et al., 2011) and increased the survival rate at 24 h post transient cerebral ischemia obtained due to bilateral carotid occlusion (Di Giacomo et al., 2012).

#### CVD Protection

The beneficial effects of dietary ACNs in the prevention of CVDs were shown by several epidemiological studies. Preventive effects of dietary ACNs concerning hypertriglyceridemia, hypercholesterolemia (total cholesterol and LDL-cholesterol) and platelet hyperactivity were reported in rat models of atherosclerosis induced by high-fat or high-fructose diets (Guo et al., 2007; Salgado et al., 2010; Yang et al., 2011) (**Figure 2**). In addition to their protective effect against atherosclerosis, ACNs also have protective effects at cardiac level against oxidative stress observed in ischemia/reperfusion condition. In rats fed with ACN-rich corn, the cardiac tissue damaged by ischemia was reduced for 39% when compared to rats fed with ACN-free corn (Toufektsian et al., 2008).

Similar results on CVD-related biomarkers were obtained in several human intervention studies on patients taking berries or purified ACNs, showing significantly increased HDL-cholesterol, reduced LDL-cholesterol, triglycerides, blood pressure, flow-mediated dilation and inflammatory markers (García-Conesa et al., 2018; Luís et al., 2018).

Higher ACN intake from berries significantly lowered to 32% the risk of myocardial infarction in young and middle-aged women (Cassidy et al., 2013; Cassidy et al., 2016) and decreased the incidence of coronary heart disease (CHD) and CVD-related mortality (Mink et al., 2007; Mccullough et al., 2012).

Other studies suggested an inverse correlation between high ACN consumption and CVD-related risk biomarkers, such as lower arterial stiffness and blood pressure (Cassidy et al., 2011; Jennings et al., 2012), reduced levels of C-reactive protein (Sesso et al., 2007; Chun et al., 2008) and reduced overall inflammation score (Jennings et al., 2014; Cassidy et al., 2015).

The cardioprotective effect of dietary ACNs may be attributed to: increase in plasma antioxidant capacity and NO levels; reduction of LDL oxidation and platelet aggregation (Erlund et al., 2008; Basu et al., 2010b; Chiva-Blanch et al., 2012; Santhakumar et al., 2015; Zhang et al., 2016; Thompson et al., 2017a; Thompson et al., 2017b).

Cardioprotection was associated with enhanced glutathione levels in pre-ischemic myocardial and marine omega-3 levels in the blood, suggesting that a physiological dose of dietary ACNs (12 mg/kg body weight/day) increase antioxidant effects and omega-3 fatty acids synthesis (Toufektsian et al., 2011).

Consistent with these results, *ex vivo* cardiac perfusion with low concentrations of bilberry ACNs resulted in a strong cardioprotective activity following ischemia/reperfusion, as shown by the low release of LDH and decrease of incidence and duration of reperfusion arrhythmias. On the contrary, high concentrations of ACNs resulted in a cardiotoxic effect, indicating a concentration-dependent cardioprotection of bilberry ACNs (Ziberna et al., 2010).

The cardioprotective activity is mediated by direct intracellular transport of C3G by the bilirubin-specific plasma membrane transporter bilitranslocase, as demonstrated by the lack of cardioprotective activity of C3G when antibodies against bilitranslocase were used before ischemia reperfusion. This shows that the entry of C3G (or other ACNs) into the endothelium is necessary to interact with intracellular targets and trigger an antioxidant response in cells or isolated organs (Ziberna et al., 2012).

#### CVDs: Brief Data on Pathophysiology

CVDs including diseases of the heart, blood vessels and the cerebrovascular system, are the killer number one, accounting for 31% of deaths worldwide (Canon, 2013).

Among CVDs, myocardial infarction, ischemic heart disease and stroke are primarily caused by atherosclerotic plaques, progressively growing and causing arterial stenosis or obstruction or eventually plaque rupture and thrombus formation (Sharifi-Rad J. et al., 2020). The process of atherosclerosis is mainly caused by oxidized low-density lipoproteins (oxLDL), typically associated to elevated plasma total- and LDL-cholesterol levels. LDL particles adhere to arterial walls and enter into the intima where they are retained and where resident macrophages and macrophages originating from circulating monocytes which bind to adhesion molecules and also enter the intima phagocytize them. Macrophage activation involves ROS production, which in turn triggers the activation of NF-κB and thereby proinflammatory cytokines and adhesion molecules causing also endothelial dysfunction. As a consequence, endothelial cells express on their surface intercellular adhesion molecule-1 (ICAM-1) and vascular adhesion molecule-1 (VCAM-1), thus attracting monocytes from the bloodstream. Once in the intima, monocytes differentiate into macrophages and massively phagocytize oxLDLs, thus becoming “foam cells”, recruiting other monocytes by releasing the chemokine monocyte chemoattracting protein-1 (MCP-1) and activated macrophages secrete proatherogenic growth-factors and cytokines.

The progression of atherosclerotic plaques involves further lipid accumulation from LDL particles, but also migration of smooth muscle cells from media to intima and their proliferation, collagen deposition, which then form a fibrous cap which covers the lipid core of the plaque. In advanced lesions, macrophage foam cells undergo apoptosis but are not effectively cleared by macrophages (defective efferocytosis), initiating a secondary cellular necrosis process and overtime to the formation of a necrotic lipid core (Moore and Tabas, 2011).

Inflammatory cells at the shoulders of the plaque cause the release of collagenases and elastase from the foam cells which might cause rupturing of the atheroma fibrous cap, platelet aggregation and thrombus formation.

Cardioprotective Mechanisms:

- Prevention of atherosclerosis using apolipoprotein E (apoE)-deficient mice, a model characterized by hypercholesterolemia and plaques on aorta with morphological features similar to human advanced atherosclerotic lesions,
- those directed toward cardioprotection using Langendorff ischemia/reperfusion injury and drug-induced cardiotoxicity and/or myocardial infarction.

##### In Vivo Studies

Concerning the apo E-deficient mice, supplementation of ACN-rich diet, extracts or ACN metabolites (e.g., protocatechuic acid) showed a significant decrease in the formation of atherosclerotic plaques (Xia et al., 2003; Xia et al., 2006; Mauray et al., 2009; Wang et al., 2010; Jiang et al., 2017; Joo et al., 2018), associated to an improvement of dyslipidemia, such as increased HDL-cholesterol (Xia et al., 2003), reduced triglycerides (Xia et al., 2006; Jiang et al., 2017), reduced total cholesterol and LDL-cholesterol in serum (Xia et al., 2003; Xia et al., 2006; Jiang et al., 2017; Joo et al., 2018) and in plaques on aorta (Xia et al., 2003; Wang et al., 2010).

In apoE-deficient mice, ACN supplementation protects LDL-cholesterol from oxidation, as proven by reduced levels of serum anti-oxLDLs (Xia et al., 2003) and of lipid peroxidation markers, malondialdehyde (MDA) and F2-isoprostane (Wu et al., 2010; Jiang et al., 2017). The reduced formation of oxLDLs may be partly accomplished by a direct scavenging activity of ACNs embedded in cellular membranes or in the cytosol of endothelial cells, where they are transported by a bilitranslocase transporter (Youdim et al., 2000; Youdim et al., 2002; Maestro et al., 2009), but this may more probably result from an increase of gene expression and activity of antioxidant enzymes, such as SOD1, SOD2, glutathione reductase (GR), thioredoxin reductase 1 (TrxR1), paraoxonase 1 (PON1) in the aorta (Wu et al., 2010) and serum SOD1, GPx as well as CAT (Jiang et al., 2017). Lower levels of oxLDLs resulted in lower expression of VCAM-1 and ICAM-1 in aorta (Wang et al., 2010; Wu et al., 2010) and lower inflammatory response, reduced activation of NF-kB and iNOS gene expression and protein levels in the aorta (Xia et al., 2003; Xia et al., 2006; Wang et al., 2010), reduced leukocyte infiltration and circulating proinflammatory cytokines (Xia et al., 2003; Joo et al., 2018).

Daily supplementation of a physiological dose of C3G (10 mg/kg body weight/day) for one week prior to surgery and eight weeks post-surgery in a rat model of myocardial infarction was able to significantly prevent cardiac dilation and improve cardiac function until four weeks after myocardial infarction. Nevertheless it was unable to sustain this cardioprotection since cardiac dysfunction was not significantly improved after 8 weeks (Raj et al., 2017).

Physiological doses of ACNs were also effective against cardiotoxicity induced by the chemotherapeutic drug doxorubicin (Dox) since in mice fed with dietary ACNs from purple corn treated with Dox, medium-term but not long-term survival was improved, and Dox-induced cardiac histopathological alterations were prevented when compared to animals fed with ACN-free diet from yellow corn (Petroni et al., 2017).

Decrease of the cardiac injury induced by cyclophosphamide, another widely used chemotherapeutic drug, was also achieved with a low dose of an ACN-rich extract from blueberry (20 mg/kg body weight/day) and it correlated to the antiinflammatory and antioxidant effects of ACNs (Liu et al., 2015). On the other hand, high concentrations of ACNs (200-250 mg/kg body weight/day) were found to be effective in experimental models of myocardial infarction induced by isoproterenol, which is known to cause an extensive oxidative damage, associated to degradation and subsequent exhaustion of enzymatic antioxidants (SOD and CAT), causing formation of free radicals and severe lipid peroxidation (Jana et al., 2017; Wei H. et al., 2017).

### Antiobesity and Antidiabetic Effects

Obesity is the result of the accumulation of adipose tissue, and it causes many metabolic disorders. A healthy lifestyle and a diet rich in ACNs have beneficial antiobesity effects (Parveen et al., 2019; Salehi et al., 2019a).

One mechanism by which dietary ACNs could act as antiobese effect is the increase of energy expenditure. Berries containing ACNs (petunidin 33% and malvidin 57%) were effective to lower HFD induced metabolic damage by increased energy expenditure. In adipose tissue, a reduction in mitochondrial respiration and dissipation of the mitochondrial proton gradient (proton leak) were also reported (Skates et al., 2018). Malvidin decreases the lipopolysaccharide (LPS)-induced NF-κB, activation of poly ADP-ribose polymerase, MAPK, depolarization of mitochondria, and generation of ROS, (Bognar et al., 2013).

Another way to spend the energy is by changing thermogenesis. The upregulation of regulating uncoupling proteins (UCP1 and UCP2), in brown and white adipose tissue respectively, suppress fat accumulation in adipose tissue in case of increased dietary consumption of black soybean seed. (Kanamoto et al., 2011).

The role of dietary ACNs in AMPK modulation is very interesting.

AMP-activated protein kinase (AMPK) is one of the main regulators of energy balance. AMPK can modulate the energetic expenditure and fat accumulation in many ways: i) increasing mitochondrial biogenesis, ii) reducing lipid metabolism and triglyceride synthesis, iii) increasing fatty acid oxidation, iv) reducing hypertriglyceridemia and triglyceride storage in muscles and liver and by regulating the food intake. It seems that dietary ACNs induce AMPK activation by increasing its phosphorylation (Hardie and Ashford, 2014; López, 2018).

ACNs from bilberry extract also increased AMPK activity in skeletal muscle and the liver. In skeletal muscle, AMPK activation stimulated the upregulation of glucose transporter 4 (GLUT4), resulting in an increased glucose uptake and utilization. In the liver, AMPK activation decreased glucose production, improving hyperglycemia. A decrease in liver lipid content and serum lipoproteins was achieved through upregulation of peroxisome proliferator-activated receptor (PPAR)α and acyl-CoA oxidase (Takikawa et al., 2010). ACNs also improved chronic diabetic complications and insulin resistance (Guo and Ling, 2015).

Dietary ACNs can also affect lipid metabolism.

The key molecules of lipid metabolism are fatty acid synthase (FAS) and sterol regulatory element-binding proteins (SREBPs). ACNs from different dietary sources could downregulate mRNA and protein levels of FAS and SREBP1, reducing hyperglycemia and inhibiting hepatic lipogenesis (Tsuda et al., 2003; Hwang et al., 2011; Qin and Anderson, 2012; Park et al., 2015; Wu et al., 2016a).

ACNs from black soybeans were effective in preventing obesity even in normal conditions by hypothalamus modulation. A healthy diet showed a decrease in body weight and food intake when receiving daily intra-gastric administered ACNs (Salehi et al., 2020b). These effects seem to be mediated by neuropeptide Y and c-amino butyric acid receptor (GABAB1R) in the hypothalamus (Badshah et al., 2013). ACN-rich extract from black soybean decreased saturated, monounsaturated and n-6 polyunsaturated fatty acid levels in subcutaneous (but not visceral) fat. Since these long-chain fatty acids play a role in inflammation regulation, their reduction could help in suppressing inflammation in obese subjects (Sato et al., 2015)

Finally, ACNs seem to affect gut microbiota as well. The change in microbiota in obese people might contribute to the development of obese-related metabolic disorders. A recent review examined the role of ACNs in obesity regulation by gut microbiota modulation (Jamar et al., 2017)

Considering the antidiabetic role of ACNs, they provide protection to pancreatic β cells (INS-1) against H_2_O_2_-induced necrosis and apoptosis in a time- and concentration-dependent way. These substances in β cells and primary islets also upregulate the HO-1 gene expression and activate ERK1/2 and PI3K/Akt signaling, while ERK1/2 and PI3K inhibitors partially decreased ACN-mediated induction of HO-1 (Zhang et al., 2011).

The supplementation with a daily intake of 4 cups of freeze-dried strawberry beverage during 8 weeks in 27 diabetic subjects caused a reduction in total and LDL-cholesterol levels as well as inhibition of VCAM-1 circulating levels (Basu et al., 2010a).

The simultaneous use of ACNs and apple polyphenols in five postmenopausal women and 20 men showed the initial postprandial glycemic response. Both substances suppressed the early reactions (0–30 min) of plasma glucose and insulin, and reduction of postprandial glycemia. Insulin and incretin excretion were reduced as the secondary results (Castro-Acosta et al., 2017). ACNs consumption during 12 weeks also modulated the lipids and glucose-metabolism, and had antioxidant and antiinflammatory effects l in 37 humans with metabolic syndrome (Kim et al., 2018). In addition, ACNs rich beverages lowered the concentrations of interferon-γ (IFN-γ) and urinary level of 8-isoprostane (Kim et al., 2018).

Wu et al. (2013) have found that ACNs, including cyanidin-3-rutinoside, cyanidin-3-glucoside, and pelargonidin-3-glucoside significantly inhibit the body weight gain, reduction in IR, adipocytes size, decrease lipid accumulation, and reduce leptin secretion. ACNs improved glucose tolerance, enhanced insulin sensitivity and decreased hepatic accumulation of lipids *via* modulating the AMPK activity and lipid metabolism-associated gene expression (Overall et al., 2017).

### Obesity: Brief Data on Pathophysiology

The number of obese people is dramatically increasing worldwide, affecting every year an increasing number of adults, but also children and adolescents (Xie et al., 2018). Obesity is closely related to a decrease in life expectancy and an increase in healthcare expenditures, it is a risk factor for many diseases such as some types of cancer, diabetes mellitus and CVDs (Swinburn et al., 2015).

The localization of the accumulated fat is crucially important: intraabdominal fat is mainly responsible for the development of the metabolic syndrome (MS), which is defined as the combination of impaired glucose tolerance or diabetes mellitus, insulin resistance, high blood pressure, atherogenic dyslipidemia, and obesity (Engin, 2017).

The effects of ACNs on MS have been recently highlighted (Naseri et al., 2018; Xie et al., 2018).

ACNs Antiobesity Mechanisms:

- Increase in energy expenditure, and regulation of lipid metabolism
- reduction of fat absorption
- suppression of food intake
- gut microbiota modification.

#### *In Vitro* Studies

In the treatment of LPS-activated human umbilical vein endothelial cells (HUVECs), pelargonidin inhibited LPS-induced barrier disruption, migration of neutrophils to human endothelial cells, and expression of cell adhesion molecules (CAMs) and adhesion.

Blueberry ACN extract (malvidin, malvidin-3-glucoside, and malvidin-3-galactoside) has effects on high glucose-induced injury in human retinal capillary endothelial cells (HRCECs) by multiple pathways such as enhancement of cell viability, reduction of ROS, suppression of Nox4 expression, increase in enzyme activity of CAT and SOD, inhibition of Akt pathway, reduction of VEGF level, suppression of high glucose-induced intercellular adhesion molecule-1 and NF-κB, (Huang et al., 2018). The administration of 300 µM H_2_O_2_ in WI-38 human diploid fibroblasts showed enhanced lipid peroxidation, lowered cell viability, and shortened cells lifespans. In contrast, cyanidin supplementation suppressed oxidative stress *via* cell viability enhancement and lipid peroxidation inhibition. Cyanidin treatment also enhanced the cells life spans, decreased the NF-κB expression at mRNA and protein level, as well as iNOS, and COX-2 (Choi et al., 2010).

Recent studies showed that pelargonidin inhibited LPS-induced hyperpermeability and leukocytes migration. Furthermore, suppression of activation of NF-κB and production of TNF-α, IL-6, and ERK1/2 by LPS were reported. In addition, pelargonidin resulted in suppressing LPS-induced lethal endotoxemia (Lee et al., 2019).

#### *In Vivo* Studies

ACNs inhibit fat accumulation in mice: purple corn extract and purified ACNs from blueberries or strawberries can prevent body fat accumulation and obesity induced by a HFD in C57BL/6J mice (Tsuda et al., 2003; Howard et al., 2008).

Increased AMPK activity has been shown in 3T3-L1 cell line treated with C3G (Guo et al., 2012), in rats treated with black carrots extract (Park et al., 2015) and in obese mice fed with ACNs from purple sweet potato (Hwang et al., 2011). In these studies, AMPK activation was accompanied by a lack of increase in LDL-cholesterol and triglycerides but with an improved serum lipids profile and inhibited accumulation of triglycerides in the liver (Hwang et al., 2011; Park et al., 2015).

Pure ACNs and ACN extract were able to reduce the body and liver weight, the triglycerides accumulation in the liver and the adipocyte size in mice treated with HFD (Jayaprakasam et al., 2006; Zhang et al., 2013; Park et al., 2017).

Blueberry-derived ACNs were effective in reducing body weight and serum glucose and in improving lipid profile in high-fat-fed mice (Wu et al., 2016a), while ACNs derived from adzuki bean decreased lipid accumulation and triglyceride/cholesterol levels in mice fed with high-fat and high-cholesterol diet (Kim et al., 2016).

A very recent study analyzed the effect of ACNs supplementation from Sango sprout juice (SSJ) in obese rats. The results showed that supplementation of SSJ is more effective causing positive effects on liver, ileum and prostate when compared with a switch from a HFD to a regular. Moreover, the SSJ supplementation together with the diet switch is more effective (in respect to a simple diet switch) in opposing the caecal *Enterococcus* decrease and the *Clostridium perfringens* increase registered in obese animals. These results demonstrate a potential therapeutic role of ACNs in obese-induced intestinal dysbiosis (Vivarelli et al., 2018).

The adipose tissue secretes various adipocytokines, i.e., leptin, adiponectin, and resistin that causes obesity to be a metabolic disease.

Supplementation of ACNs-rich grape–bilberry juice to experimental rats lowered the concentrations of cholesterol, triglycerides, resistin, and leptin. This supplementation also decreased the saturated fatty acids and increased polyunsaturated fatty acids in plasma (Graf et al., 2013). ACNs decreased the secretion of adipocytokines (adiponectin and leptin) and increased lipoprotein lipase (LPL), PPARγ, UCP2, and adipocyte fatty acid-binding protein (aP2) expression in isolated rat adipocytes (Tsuda et al., 2004). Furthermore, ACNs-rich extracts have an improving effect against D-galactose-induced senescence in a mice model *via* lowering the uric acid level (Lu et al., 2014). ACNs have also beneficial affects against prostatic hyperplasia (Jang et al., 2010). In sickle cell disease, ACNs stabilize the erythrocyte membrane and suppress the hemoglobin polymerization (Mpiana et al., 2010). Supplementation of ACNs to C57BL/6J mice caused a significant reduction in concentrations of serum cholesterol, insulin resistance (IR), lipid accumulation and leptin secretion. These substances also have the potential to change MAPK and NF-κB stress signaling pathways (Wu et al., 2013).

In another study reported by Seymour et al. (2011), they investigated the administration of blueberry ACNs on white adipose tissue (WAT) and skeletal muscle in Zucker-fatty rats at the concentration of 2% (wt/wt). They have found that ACNs decreased the intraperitoneal fat weight and increased PPAR activity. Likewise, administration of ACNs at concentration of 8% (wt/wt) during 8 weeks decreased the inflammatory markers, enhanced the blood adiponectin levels, decreased the adipose tissue hypertrophy, hepatic steatosis, and insulin resistance in WAT and improved dyslipidemia (Vendrame et al., 2013).

Consistent with this, supplementation of mice under HFD with an ACNs-rich extract from purple corn resulted in lower recruitment and proliferation of macrophages into crown-like structures in the adipose tissue caused by a suppression of NF-kB signaling. Besides attenuating adipose tissue inflammation *in vivo*, ACNs also showed a long-lasting reprogramming of adipose tissue macrophages and adipocyte profiles toward the antiinflammatory phenotype (Tomay et al., 2019). Alteration in genes expression involved in lipid metabolism protect the induced fatty acid oxidation, and decrease the *in vivo* biosynthesis of fatty acids and cholesterol (Wu et al., 2013; Song et al., 2016).

### Antioxidant and Antiinflammatory Effects

ACNs extracts from different natural sources were able to decrease the oxidative stress. (**Figure 2**) Dietary ACNs are known to be more effective antioxidants than vitamins E and C (Rice-Evans et al., 1997). ACNs can modulate the antioxidant defense mechanisms, activate antioxidant enzymes and promote glutathione synthesis. They are capable of chelating metal ions, such as iron and copper, thus reducing the production of free radicals by Fenton and other reactions (Jomova and Valko, 2011).

Several studies showed that ACNs activate nuclear factor erythroid 2-related factor 2 (Nrf2) and the antioxidant enzymes, such as superoxide dismutase (SOD), catalase (CAT), glutathione peroxidase (GPx) as well as directly enhance their enzymatic activity (Speciale et al., 2011; Speciale et al., 2013).

ACNs are also able to oppose the harmful action of toxic agents. ACNs from blueberry extract suppress the effects of acrylamide, attenuating ROS overproduction and glutathione depletion in liver. They were also effective in inhibiting cytochrome P450 2E1 (CYP2E1) protein expression in acrylamide-treated mice. CYP2E1 is the first protein involved in acrylamide epoxidation that was shown to cause different toxic effects (Zhao et al., 2015). The inhibition of CYP2E1 protein is also involved in the ACN-mediated protection of ethanol- and ROS-mediated damage. Ethanol, in fact, activates CYP2E1 that causes ROS production and antioxidant defense mechanisms impairment; ACNs in *Gynura bicolor* (Roxb. ex Willd.) DC. restored the glutathione content and decreased the ROS and glutathione disulfide levels in livers of ethanol-treated mice by reduction of CYP2E1 activity (Yin et al., 2017).

#### Inflammation: Brief Data on Pathophysiology

Oxidative stress occurs when reactive oxygen and nitrogen species production (ROS and RNS) exceeds the antioxidant mechanisms of cells or tissues, thus resulting in damage of macromolecules (i.e. proteins, lipids and DNA) in chronic diseases and aging process. Acute inflammation is the primary response against injury and pathogens, and it is usually followed by the resolution of inflammation (Salehi et al., 2019b). Chronic inflammation resulting from the failure of resolution is reported to promote the progression of many chronic diseases, such as CVDs, neurodegenerative diseases, diabetes mellitus and cancers (Pham-Huy et al., 2008; Salehi et al., 2018; Sharifi-Rad et al., 2018) (Medzhitov, 2008; Docea et al., 2020).

Antiinflammatory Molecular Mechanisms:

- ACNs suppress the activation of the nuclear factor kappa B (NF-κB), a transcription factor regulating many genes in inflammatory response, such as inducible NO synthase (iNOS), cycloxygenase-2 (COX-2) and proinflammatory cytokines [tumor necrosis factor-α (TNF-α), interleukin (IL)-1β and IL-6] (Tsuda et al., 2002; Poulose et al., 2012).
- ACNs inhibit the mitogen-activated protein kinase (MAPK) signaling cascade involving p38, JNK, and ERK, also inducing suppression of proinflammatory cytokines, iNOS and COX-2 (Hou et al., 2005).
- ACNs directly inhibit COX-1 and COX-2 enzymes and as a consequence of this, the production of prostaglandin E2 (PGE2) (Graf et al., 2013; Hassimotto et al., 2013).
- ACNs suppress LRR, NACHT and PYD domains-containing protein 3 (NLRP3) inflammasomes by activation of Nrf2 and the thioredoxin-1/thioredoxin-interacting protein (Trx1/TXNIP) inhibitory complex (Cui et al., 2018; Hou et al., 2018). The NLRP3 inflammasome is a multimeric protein complex that initiates an inflammatory form of apoptosis, by triggering the release of proinflammatory cytokines IL-1β, IL-18 and caspase-1 and has been implicated in several diseases (Yang et al., 2019).

##### In Vivo Studies

The antioxidant activity of ACNs is well known for decades. (Pojer et al., 2013; Khoo et al., 2017). ACN extracts and pure ACNs increase the hepatic and serum levels of SOD and CAT in mice and rats while decreasing free radicals’ generation (Chiang et al., 2006; Roy et al., 2008).

A purple sweet potato color (PSPC) prevents the HFD-induced endoplasmic reticulum-mediated oxidative stress in mice liver. PSPC improved the hepatic redox state of mice treated with HFD by suppressing ROS production and by restoring the glutathione content and the activity of antioxidant enzymes (Zhang et al., 2013).

Similar results were observed in mice under HFD supplemented with ACNs from cherry or mulberry. Significant increases in SOD and GPx activities were detected. It was shown that monoglycoside ACNs might have higher antioxidant effects than di-glycoside or tri-glycoside ACNs (Wu et al., 2016b). Cyanidin 3-glucoside (C3G) was efficient in reducing the oxidative stress induced by lipid peroxidation, neutrophiles infiltration, and hepatic steatosis in diabetic mice. C3G increased glutathione synthesis by the induction of the glutamate-cysteine ligase catalytic subunit mediated by protein kinase A (PKA) and cAMP-response-element binding protein (CREB) (Zhu et al., 2012).

ACNs can improve ROS-caused damage in the brain. ACNs from Korean black bean inhibited ROS production induced by ethanol in the hippocampus of the postnatal rats (Shah et al., 2015). ACNs from black soybean suppressed neuroinflammation and neurodegeneration caused by oxidative stress and ROS increase in the cortex of adult mice (Khan et al., 2016).

#### ACNs as Natural Compounds With Potential Anticancer Properties

ACNs have attracted interest in the last several decades as potential antitumoral agents (Pojer et al., 2013; Lin et al., 2017).

There are several pathways involved in cancer, and some of them are not yet explained. Uncontrolled cell proliferation, resistance to apoptosis and migration are the main characteristics of tumor cells, which can be due to malfunctioning of Notch, Wnt/β-catenin, NF-κB and MAPK pathways (Dreesen and Brivanlou, 2007). Dietary berry ACNs can modulate the levels of Notch1 and Wnt1 proteins and their downstream mediators. In particular, ACNs mixture showed an enhanced reduction of all the proteins when compared with the single purified ACNs, indicating that some pathways may overlap in the induction of cell growth inhibition (Kausar et al., 2012).

The ACNs-mediated cancer prevention and inhibition mainly includes pathways involved in cell survival, proliferation, apoptosis control and inflammation. PI3K/Akt, NF-κB and COX-2 signaling and activity are the most studied mechanisms.

Although inflammation and oxidative stress play an important role in cancer progression (Kristo et al., 2016), it seems that ACNs, and not anthocyanidins, are responsible for the interaction with different molecules. In contrast, the glucosidic part may reduce the beneficial properties and interactions (Song et al., 2012).

NF-κB is downstream of the PI3K/Akt pathway, which is important for cell survival and proliferation. Still, if activated, it can cause deregulation of cell growth, malignant transformation and, often, therapy resistance (Hennessy et al., 2005). NF-κB has been indicated as the mediator between chronic inflammation and cancer. It can regulate tumor angiogenesis, metastatic process and apoptosis inhibition (Salehi et al., 2019d).

It has been suggested as a possible target in cancer therapy, even if its prolonged inhibition can cause deleterious effects (Xia et al., 2014). Its expression, together with COX-2, another factor involved in inflammation, and PI3K/Akt pathway, are modulated by ACNs, leading to reduced inflammatory response and cancer progression due to reduced proliferation (Song et al., 2012; Peiffer et al., 2014; Medda et al., 2015; Choi et al., 2016; Fragoso et al., 2018).

The main problem with chemotherapy is that some cancers can develop resistance to treatment after several months. Angiogenesis, metastasis progression and cell migration can severely worsen the patient status and compromise the success of chemotherapy (Mishra et al., 2018). Trastuzumab (Herceptin^®^) is a recombinant humanized monoclonal antibody that is targeted against human HER2 tyrosine kinase receptor, and it has been successfully used to treat patients with HER2-positive breast cancer. However, some trastuzumab-treated patients, who initially responded well, showed disease progression within a year after the end of the treatment (Li X. et al., 2016). C3G can enhance trastuzumab (Liu et al., 2014; Li X. et al., 2016).

Potential Mechanisms of Anticancer Properties:

- Inhibition of tumor growth
- promoting apoptosis and autophagy of malignant cells
- modulating signal transduction pathways
- inhibition of angiogenesis and metastatic migration
- antiinflammatory and antioxidant properties

##### In Vitro Studies

Delphinidin, a substance belonging to ACNs, dose-dependently suppresses cell proliferation and invasion, it induces apoptotic cell death and autophagy in human epidermal growth factor receptor (HER)-2 positive breast cancer MDA-MB-453 and BT474 cells. Moreover, it causes induction of autophagy *via* inhibiting mTOR signaling pathway and activation of the AMPK signaling pathway in HER-2 positive breast cancer cells (Chen J. et al., 2018).

In a recent study by Zhou et al. (2017), they investigated whether ACNs from black rice could have suppressive effect on HER-2 positive human breast cancer cell metastases in different human cancer cells lines—MCF-7, MCF-10A, and MDA-MB-453 cells. ACNs significantly inhibited the migration and cell invasion, lowered the migration distance of HER-2 positive human breast cancer cells, phosphorylation of cSrc, FAK, and p130C, lowered the levels of mesenchymal markers (fibronectin, vimentin), decreased the interaction between HER-2 and FAK, FAK and cSrc, and inhibited the epithelial-mesenchymal transition (Zhou et al., 2017).

The antitumor effect of ACNs in human hepatoma cells (SMMC-7721) and the murine hepatoma cells (H22) studies were also highlighted. ACNs significantly suppressed the cell growth, blocked the cell cycle in G2/M phase, induced DNA damage and induced apoptosis

C3G is a strong anticancer agent in different human breast cancer cell lines such as Hs‐578T and MDA‐MB‐231 cells. It achieves it by inhibiting the vascular endothelial growth factor (VEGF) expression and secretion, by decreasing the activator of transcription 3 (STAT3) and signal transducer expression at both mRNA and protein level. Additionally, induction of miR-124 expression was also reported after ACNs treatment (Salehi et al., 2019e).

A study conducted by Mazewski et al. (2018) was looking whether ACNs have inhibitory effects in concentrations (IC_50_) such as 0.9–2.0 mg/ml on human colon cancer cells (HT-29 and HCT-116) proliferation by inducing apoptotic cell death, decreasing the levels of antiapoptotic proteins (cIAP-2, survivin, XIAP), arresting cells in G1 phase as well as having a tyrosine kinase inhibitory potential. They have confirmed all these effects.

In another study, ACNs in a human gastrointestinal model (Colonic Caco-2 cancer cells and nontumorigenic colonic CCD-112CoN cells) cause cytotoxicity and lower cell viability (Kubow et al., 2017). Kuntz et al. (2017) showed that ACNs decreased cells migration, reactive oxygen production, NF-κB as well as matrix metalloproteinase (MMP)-2 and MMP-9 mRNA expression levels in different pancreatic cancer cells (PANC-1 and AsPC-1).

In another study conducted by Giampieri et al. (2018), ACNs have cytotoxic effects on hepatocellular carcinoma cells in a dose/time-dependent manner *via* enhancing cellular apoptosis and impairing mitochondrial functionality.

Cyanidin 3-rutinoside can inhibit the motility of RKO human colon cancer cells, as demonstrated by a wound-healing assay (Fragoso et al., 2018). Moreover, VEGF-induced angiogenesis was strongly inhibited by black raspberry extract on two organ-specific primary cells [i.e. human intestinal microvascular endothelial cells (HIMEC) and human esophagal microvascular endothelial cells (HEMEC)], isolated from surgically resected human intestine and esophagus (Medda et al., 2015).

##### In Vivo Studies

Xenograft tumors’ dimension in nude mice is severely reduced by pure C3G, ACNs from black soybean and by berry anthocyanidins mixture (C3G, malvidin, peonidin, petunidin, and delphinidin) (Kausar et al., 2012; Chen et al., 2015; Ha et al., 2015).

In different colorectal cancer models [DMH and TNBS-induced colitis-associated carcinogenesis in rat, azoxymethane (AOM)/dextran sodium sulfate (DSS) mouse model, APC^MIN^ mice] the administration of ACNs sources, like açai pulp (Choi et al., 2016; Fragoso et al., 2018), purple sweet potato extract (Asadi et al., 2017) and bilberry extract (Lippert et al., 2017), resulted in reduced tumor growth, slower tumor development and reduced number of adenoma.

ACN extract of roselle (*Hibiscus* ACNs) was supplemented in the diet of the rat model of N-methyl-N-nitrosourea (NMU) -induced leukemia, and it significantly reduced the elevated aspartate aminotransferase (AST) and alanine aminotransferase (ALT) levels in serum and blood, and prevented NMU-induced leukemic cell infiltration and subsequent tissue damage (Tsai et al., 2014). Not only ACNs but also their metabolites can prevent tumor growth (Peiffer et al., 2014). In the same study protocatechuic acid, a major metabolite of blackberry ACNs, was able to reduce esophagal carcinogenesis in N-nitroso methyl benzylamine (NMBA)-induced rats carcinogenesis. They also provoked the death of tumor cells, inhibited tumor growth, and improved the survival status of H22 tumor-bearing mice. These effects were associated with an increase of the antioxidant mechanism (SOD, GPx, and glutathione) and a decrease of the lipid peroxidation (MDA). The levels of immune cytokines, including IL-2, IFN-γ, and TNF-α, were also regulated by ACNs (Zhou et al., 2018).

In AOM/DSS-treated C57BL/6J mice, ACNs enhanced the decreased probiotics (*Eubacterium rectale*, *Faecalibacterium prausnitzii*, and *Lactobacillus*) and the enhanced pathogenic bacteria (*Desulfovibrio* sp. and *Enterococcus* spp). These substances caused demethylation of the secreted frizzled related protein 2 (SFRP2) gene promoter, resulting in an increased expression of SFRP2, both at the mRNA and protein levels. In addition, they also down-regulated the DNMT31 and DNMT3B, and p-STAT3 expression (Chen L. et al., 2018).

Topical application of C3G can also reduce COX-2 levels and NF-κB activation in the skin of UV-B exposed mice (Pratheeshkumar et al., 2014). The PI3K/Akt pathway can also induce mTOR activation (Hennessy et al., 2005). In a study performed on thyroid cancer cells (SW1736 and HTh-7), mulberry ACNs induced apoptosis by severely enhanced autophagy caused by the suppression of Akt/mTOR signaling (Long et al., 2018). ACNs *in vitro* induced apoptosis in cancer cells by the increase of cleavage/activation of caspase-3, p53 expression, and Bax/Bcl-2 ratio together with increased NAD^+^/NADH ratio (Kausar et al., 2012; Ha et al., 2015).

## Discussion, Therapeutic Limitations, and Clinical Pitfalls

There is a great interest in general public in the consumption of colorful phytochemicals such as ACNs, carotenoids and flavonoids, which are present in food and dietary supplements as well as in nutraceuticals. Polyphenolic substances, including flavonoids, are one of the most important classes of natural compounds that have a remarkable biological activity (Sharifi-Rad et al., 2020a).

Phenol-derived compounds including polyphenols, flavonoids and anthocyanidins have been recognized among the most promising secondary metabolites of naturally occurring compounds with therapeutic potentials. ACNs could be directly absorbed and found in animal or human plasma, while anthocyanidins have low bioavailability. The increased number of attached sugars in the aglycon might negatively affect the binding ability of ACNs to different targets (Sogo et al., 2014).

The main limitation in clinical therapy emerge from the low bioavailability of ACNs (Czank et al., 2013), their instability at physiological pH (Kay et al., 2009) and their massive conversion into metabolites once absorbed as well as by the intestinal microbiota (Aura et al., 2005; Czank et al., 2013). These aspects suggest that ACNs mainly accomplish their role of direct scavengers in the gut.

In order to improve the bioavailability and clinical usage of ACNs, chemical modifications and new drug design, such as nanotechnology were developed (Braga et al., 2018; Pinzaru et al., 2018).

The protection and especially the controlled release of various organic molecules is achieved almost exclusively by means of encapsulated compounds.

Cyclodextrins are part of the most used class receptors in host-guest inclusion chemistry. Advantages of encapsulation in cyclodextrins of active substances biological effects are: improving bioavailability, increasing stability, reducing side effects. However, they can function as molecular signals, being able to activate the endogenous antioxidant defense mechanisms (Virgili and Marino, 2008).

Nanoencapsulation is an example of a new research possibility that allows enhancing the bioavailability and optimizing the delivery of phytochemicals (Khan H. et al., 2019), including ACNs. Advantages of nanoencapsulation are: preservation of flavor, enhancing thermal and oxidative stability of chemical compounds, overcoming the limitations of high volatility, controlling the release of substances and improving bioavailability. Nanoencapsulation is more efficient and has better encapsulation properties than microencapsulation. All of these characteristics increase the possibilities of applications of phytochemicals in food and beverages. (Wyspianska et al., 2019).

The combination of carriers, e.g., chitosan with protein, improve the capsule efficiency and functionality (Ge et al., 2019). ACNs are hydrophilic natural chemical substances and cannot cross the plasma membrane by passive diffusion. Therefore, they need a hydrophilic carrier (Walton et al., 2006b). Their bioaccessibility potential is also depending upon their mineral contents, particularly potassium. *In vitro* digestion procedures can be used to evaluate the bioaccessibility (Gomes et al., 2019). Usually are used gastric simulation and small intestinal digestion models, sometimes followed by Caco-2 cells uptake (Vaidyanathan and Walle, 2001).

The preparation parameters of nanocomplexes with chitosan hydrochloride, inulin, and carboxymethyl chitosan as carrier showed maximum ACNs retention rate, preferred particle size and high encapsulation efficiency. For instance, to increase the bioavailability, the ACN source was encapsulated with liposomal micelles. The taste, smell and color of ACNs became more acceptable to consumers with encapsulation of isotonic drinks and extracts of fruits. Besides, the beverages enriched with inulin microcapsules had also better stability during storage (Tarone et al., 2020).

The main clinical pitfall of ACNs therapeutics usage is that certain drugs interact with ACNs. Studies have shown that the cytochrome P450 enzyme, which is involved in drug metabolism, is inhibited by flavonoids. An efflux transporter called P-glycoprotein, which decreases the absorption of certain drugs, is also affected. ACNs also interact with certain nutrients. They can bind to iron, thus decreasing its absorption in the intestine. Some of ACNs also inhibit cellular absorption of vitamin C (Ayabe and Akashi, 2006).

## Overall Conclusions and Future Perspectives

ACNs are a diverse group of phytonutrients found in almost all fruits and vegetables. Along with carotenoids, they are responsible for the vivid colors of fruits and vegetables. Anthocyanidins include malvidin, pelargondin, peoidin, and cyanidin. Good sources of anthocyanidins are red, purple and blue fruits, such as pomegranates, plums, red wine and red and black grapes. Anthocyanidins are associated with heart health, antioxidant effects and help prevent obesity and diabetes.

ACNs are also widely used as natural dyes in the food industry. They have a wide range of color tones, ranging from orange to red to purple and blue, depending on the molecular structure and pH value. Interest in ACNs is not only based on their coloring effect, but also due to their health-beneficial properties. Due to the growing environmental and health problems in terms of synthetic dyes, natural dyes are an excellent alternative as an environmentally friendly dye for the food and drug industry.

ACNs are rapidly absorbed and appear in the bloodstream only few minutes after consumption. Future studies need to be planned to enable better understanding of the mechanisms by which food components achieve their effects and their pharmacokinetic characteristics. Antioxidant and antiinflammatory effects of ACNs are proven and it seems that ACNs also have an important role in CVDs, neurodegenerative diseases, diabetes and cancer. The potential of ACNs to affect mammalian metabolism is demonstrated in *in vitro* and *in vivo* studies. Dietary ACNs may be a potential regulator of obesity-derived inflammation and associated chronic diseases.

Future clinical studies, using food rich in ACNs and purified ACNs need to be performed, better understand the therapeutic potential of these antioxidant substances.

## Author Contributions

Conceptualization JS-R and DC. Validation investigation, resources, data curation, writing—all authors. Review and editing—JS-R, KP, MM, AM, ŽR, and DC. All authors contributed to the article and approved the submitted version.

## Funding

This work was supported by CONICYT PIA/APOYO CCTE AFB170007.

## Conflict of Interest

The authors declare that the research was conducted in the absence of any commercial or financial relationships that could be construed as a potential conflict of interest.
